# Epstein-Barr Virus: From Kissing Disease to Broken Heart

**DOI:** 10.7759/cureus.7704

**Published:** 2020-04-17

**Authors:** Daniel Rosas, Isaac Yepes, Jacqueline Tschanz, Minaba Wariboko, Jose D Sandoval-Sus

**Affiliations:** 1 Internal Medicine, Memorial Healthcare, Sunrise, USA; 2 Internal Medicine, Memorial Hospital West Healthcare System, Pembroke Pines, USA; 3 Cardiology, Memorial Hospital West Healthcare System, Pembroke Pines, USA; 4 Malignant Hematology & Cellular Therapy, Moffitt Cancer Center at Memorial Healthcare System, Pembroke Pines, USA

**Keywords:** epstein-barr virus infections, lymphoma, burkitt lymphoma

## Abstract

We present a case of a 59 year old female patient that presented with exertional chest pain and palpitations. A workup revealed an EKG with signs of right ventricular hypertrophy, a high Pro-BNP and 3 sets of negative troponin levels. A CT scan of the chest was negative for pulmonary embolism (PE) but revealed a nodular thickening of the atrial septum with right atrial extension encasing the right coronary artery. A CT scan of the abdomen and pelvis with IV contrast revealed several nodular foci scattered in the subcutaneous fat of the abdominal wall bilaterally. An initial transthoracic echocardiogram (TTE) revealed thickening of the interatrial septum with a mass protruding from the interatrial septum into the left atrium and a secondary pedunculated mass protruding from the interatrial septum into the right atrium with significant obstruction within the right atrium.

An ultrasound-guided biopsy of the soft tissue nodule in the right anterior abdominal wall and subcutaneous tissue showed the classical starry sky appearance pattern confirmed later to be a Burkitt lymphoma. The patient received chemotherapy and follow up CT of the abdomen and pelvis reported resolution of the soft tissue density involving the partially visualized portions of the heart.

Although rare, cardiac lymphomas should be considered in the differential diagnosis of patients with identified cardiac masses. As the initial presentation is usually composed by non-specific symptoms, a detailed clinical history can identify certain constitutional symptoms and a thorough physical exam can lead to the suspicion of cardiac structural pathology prompting the need for the appropriate chest imaging. Further characterization may need TTE or TEE which are more sensitive and specific due to the tri-dimensional and temporal quality of the imaging. Appropriate biopsy with pathology and molecular studies are of utmost importance in making an accurate diagnosis in order to select the best management for this highly aggressive malignancy.

## Introduction

The prevalence of non-Hodgkin lymphomas (NHL) involving the heart ranges between 9-24% [1]. Among these, diffuse large B cell lymphoma (DLBCL) is the most common and Burkitt Lymphoma (BL) has been reported only in extremely rare cases. BL by itself represents just 1% of all non-Hodgkin lymphomas (NHL) and has a prevalence in the general population of 0.30 per 100,000 people [2]. In patients with Human Immunodeficiency virus (HIV) this percentage can reach almost 30% [3,4]. In the current literature, most of the reported cases of lymphoma involving the heart presented in immunocompromised patients with HIV. We report a case in a middle age woman without a diagnosis of HIV and otherwise immunocompetent.

## Case presentation

A 59 year old hispanic patient presented with palpitations and pressure like retrosternal chest pain which was more pronounced with exertion, lying flat and while coughing. She also mentioned that she had some bilateral calf pain and dry cough for the last 3 weeks. She was admitted to the hospital two years ago with similar symptoms and was found to have paroxysmal tachycardia. Physical examination was remarkable for tachycardia and irregular heart sounds and pulse. She had mild wheezing as well. Laboratory work-up was negative for HIV, Pro-BNP was 665 pg/ml, three sets of troponin were negative and she had mild normocytic normochromic anemia. EKG on admission showed sinus tachycardia with right axis deviation and signs of right ventricular hypertrophy.

**Figure 1:**
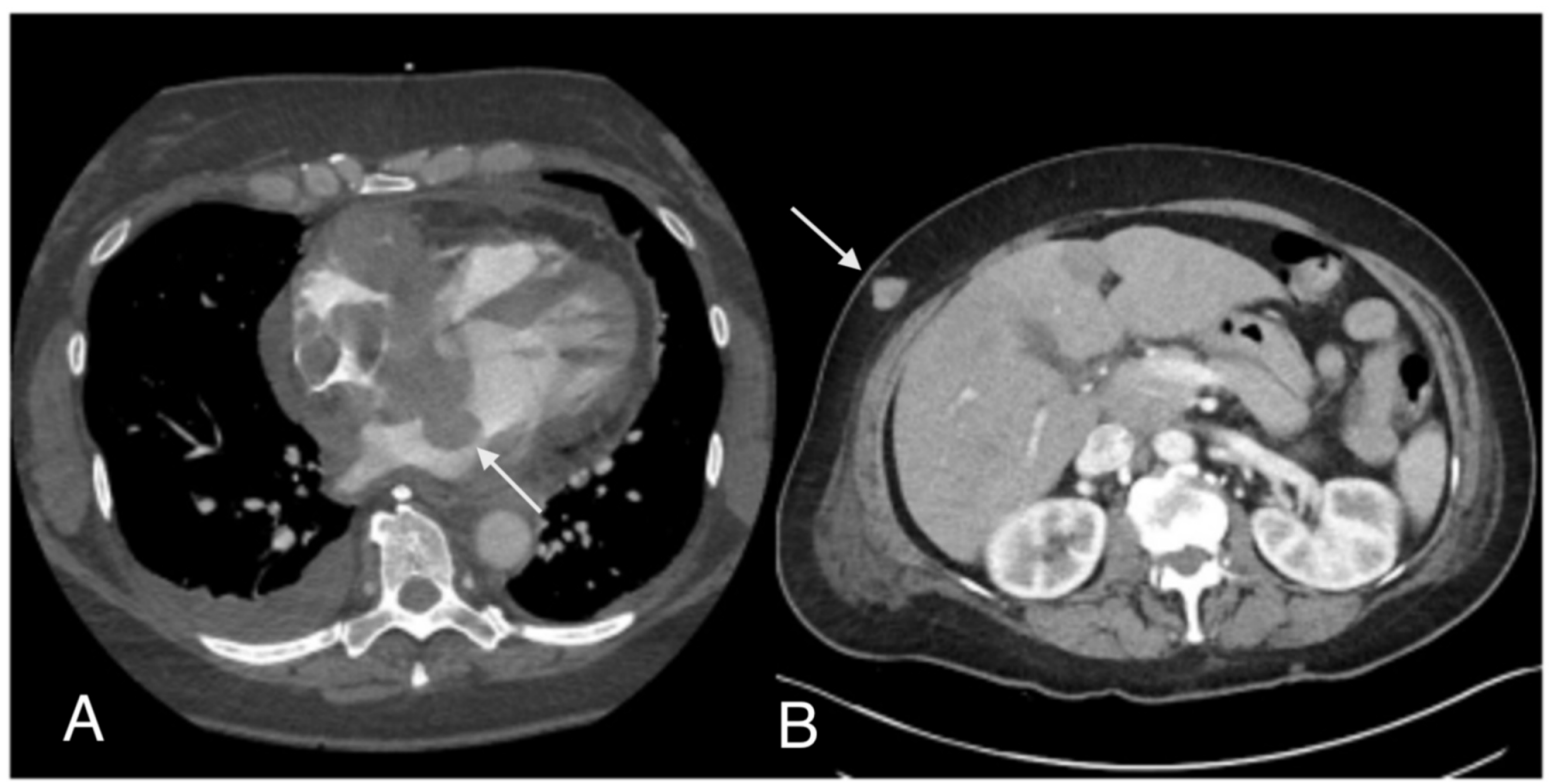
CT abdomen and pelvis with IV contrast. Left panel: Interatrial septal cardiac masses; Right panel: Abdominal wall mass

**Figure 2:**
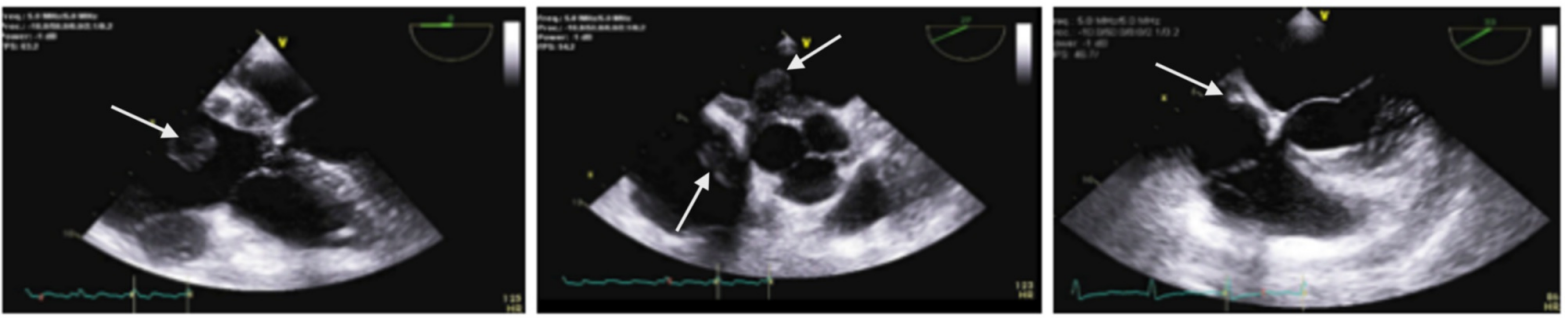
Left panel- TEE Mid esophageal view which shows infiltration of the interatrial septum and a large protruding right atrial mass. Middle panel- TEE short axis view which shows two large mobile pedunculated masses extending in the right and left atrium. Right panel- TEE mid esophageal view post chemotherapy which shows significant reduction in size of the right atrial mass; and complete resolution in the masses that once infiltrated the interatrial septum.

A computed tomography (CT) of the chest was ordered to rule out pulmonary embolism and revealed a nodular thickening of the interatrial septum extending to the right and left atria as well as a nodular soft tissue density encasing the right coronary artery (RCA) and a small pericardial effusion. Mediastinal, internal mammary and right hilar lymphadenopathy were also noticed (Figure 1A). A CT scan of the abdomen and pelvis with IV contrast revealed several nodular foci scattered in the subcutaneous fat of the abdominal wall bilaterally including a 2.0 x 1.4 cm nodular focus involving the anterolateral right abdominal wall (Figure 1B). An initial transthoracic echocardiogram (TTE) revealed thickening of the interatrial septum with a 3.6 cm x 2.6 cm mass protruding from the interatrial septum into the left atrium and a 3.1cm x 5.3 cm pedunculated mass protruding from the interatrial septum into the right atrium with significant obstruction within the right atrium identified by color flow Doppler. The left ventricle (LV) function was hyperdynamic and no valvular abnormalities were found (Figure 2).

**Figure 3:**
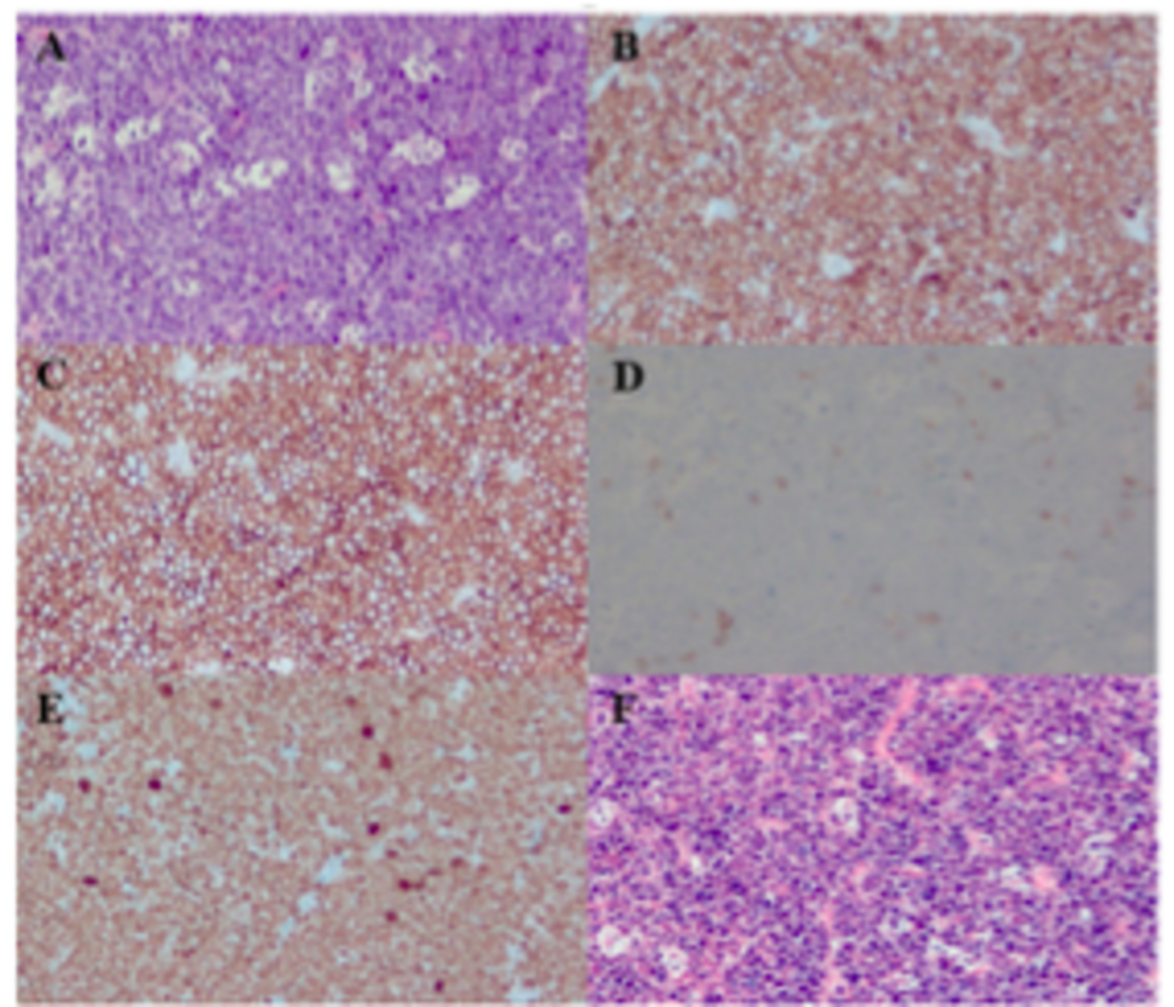
An H&E section of the right abdominal wall nodule biopsy shows a dense neoplastic lymphoid infiltrate consisting of intermediate sized lymphocytes with frequent apoptotic bodies imparting a starry sky appearance (A). The CD20 immuno-histochemical stain (B) shows the neoplastic lymphoid cells are B-cells with co-expression of CD10 (C). B-cells are negative for BCL2 (D) and a show a Ki67 proliferation index of nearly 100% (E). The in situ hybridization for EBV encoded RNA (EBER) is positive.

Initially a myocardial biopsy was intended. However, due to safety concerns and also because the cardiac mass was thought to be from the same origin as the abdominal mass, it was deferred and an ultrasound-guided biopsy of the soft tissue nodule in the right anterior abdominal wall and subcutaneous tissue was obtained. Histologic sections revealed a dense atypical lymphoid infiltrate mainly consisting of intermediate sized lymphocytes with high nuclear to cytoplasmic ratio with one to several small nucleoli with frequent apoptotic bodies imparting a starry sky appearance to the lymphoid infiltrate and mitotic figures (Figure 3). On immunohistochemical stains and immunofluorescence in situ hybridization studies (FISH), the neoplastic lymphoid cells were positive for CD45, CD20, PAX5, CD10, BCL6, CD43, and EBER, predominantly positive for c-MYC, and were negative for BCL2, MUM1, CD5, TdT, CD34, cyclin D1, CD30, and HHV8. The Ki-67 proliferation fraction was 100%. CD3 and CD5 highlight scattered T cells. CD21 did not show any follicular dendritic cell meshworks. Few scattered CD138+, MUM1+ plasma cells appeared to show a mixture of kappa and lambda positive cells by in situ hybridization. The FISH analysis of the specimen from part B (F19-785) revealed a kappa clonal B-cell population with co-expression of CD10.

A diagnosis of high risk Burkitt's lymphoma stage IV (extra nodal involvement) was made. Testing for EBV DNA was positive with qualitative analysis revealing 165,163 copies/mL. The patient was started on chemotherapy regimen of DA EPOCH-R (Etoposide, prednisone, vincristine, cyclophosphamide, doxorubicin, and rituximab). The patient was discharged 3 weeks later and a new TTE reported smaller size of the nodular masses involving the interatrial septum in the left atrium. The patient received 4 cycles of DA EPOCH-R and a follow up CT of the abdomen and pelvis reported resolution of the soft tissue density involving the partially visualized portions of the heart, pericardium and abdominal wall.

## Discussion

Burkitt lymphomas encompass only 1% of all the non-Hodgkin lymphomas and heart involvement is rare with a maximal prevalence reaching 24%. Among patients presenting with NHL involving the heart, DLBCL is the most common. Very rarely, there have been other cases of primary cardiac Burkitt lymphoma reported in the literature with a review of the literature reporting only 13 manuscripts [1,3]. Most of the cases presenting with primary cardiac lymphoma (PCL) were patients with a diagnosis of HIV with the immunocompromised state making them more susceptible to this aggressive malignancy. In our patient, a diagnosis of HIV was excluded on admission. Burkitt lymphomas have a mutation in the MYC gene and this mutation is associated with EBV activation of B-cells. EBV infection is found in 5-10% of sporadic Burkitt lymphoma and 30% of HIV associated Burkitt lymphoma [5-8].

Although a typical presentation of Burkitt lymphoma comes with abdominal pain and constitutional symptoms, a patient presenting with initial cardiac signs and symptoms like chest pain, orthopnea or signs of heart failure may warrant chest imaging and echocardiography. Most of the cases of cardiac lymphomas are found incidentally during imaging studies performed to rule out another differential diagnosis. In this case, a chest CT was performed to rule out a pulmonary embolism. Incidental cardiac masses are often benign in nature with most cases being a thrombus or vegetation. Even when malignancy is indeed suggested by imaging, cardiac metastasis from a known breast, lung or malignant melanoma are more common than primary cardiac tumors. From those being primary cardiac tumors, 90% are benign with atrial myxomas making up 50% of the cases and papillary fibroelastomas are the second most common. Primary malignant cardiac tumors are even more infrequent and represent only 25% of all primary cardiac tumors with most of those being sarcomas [5,6]. With that being said, finding a primary cardiac Burkitt lymphoma is areportable case. Chan et al, did a review of the literature and found only 22 cases reported in the English literature including their case. Only 7 of these were in patients with confirmed negative HIV status [4].

Mortality is high as reported back in 2008 by Chan et al. In their review of the literature almost 50% of the 22 cases and with the longest surviving patient reported to be alive at 36 months after the diagnosis [4]. With new chemotherapy regimens, better imaging modalities, and multidisciplinary management of these patients, early diagnosis and more effective treatment prevent these patients from getting locally advanced cardiac involvement. This includes its inherent mechanical complications due to right heart involvement.

Petrich et al. found that immune status, left ventricular involvement, the presence of extra-cardiac disease and arrhythmia were 4 factors that markedly impact survival [5]. This may correlate with how locally advanced is the cardiac involvement from the lymphoma.

The correct pathologic diagnosis is essential in order to select the appropriate systemic chemotherapy regimen, which as per the available literature is the only effective therapy for this kind of malignancy [7]. In our case, we able to avoid endomyocarial biopsy as there was a more suitable abdominal wall lesion that confirmed diagnosis. There are potential serious complications of systemic chemotherapy on the heart, including cardiac toxicity and the mechanical complications due to regression of the lesions and alteration of the surrounding cardiac structures. All these can end in cardiac free wall rupture, atrial septal defect (ASD), ventricular septal defect (VSD) or valvular apparatus alterations with ensuing valvular insufficiency. Follow up imaging with TTE or TEE allows for identification of these potential complications as well as assessment for response to chemotherapy. In our case, this follow up imaging found complete resolution of the interatrial septal nodular masses. The interdisciplinary approach with management and follow up by Hematology-Oncology in conjunction with Cardiology allows for better outcomes in the management of this aggressive and rare subset of lymphoma.

## Conclusions

Although rare, cardiac lymphomas should be considered in the differential diagnosis of patients with identified cardiac masses. Although the initial presentation is usually composed by random symptoms, a detailed clinical history can identify certain constitutional symptoms and a thorough physical exam can lead to the suspicion of cardiac structural pathology. These might lead to findings prompting the need for the appropriate chest imaging. Most of the cardiac masses are incidentally identified in imaging studies. Further characterization may need TTE or TEE which are more sensitive and specific due to the tridimensional and temporal quality of the imaging. Appropriate biopsy with pathology and molecular studies are of utmost importance in making an accurate diagnosis in order to select the best management for this highly aggressive malignancy.
